# High-resolution spin-polarized scanning tunneling spectroscopy using a functionalized superconducting tip

**DOI:** 10.1093/jmicro/dfag001

**Published:** 2026-01-16

**Authors:** T Machida

**Affiliations:** RIKEN Center for Emergent Matter Science, 2-1 Hirosawa, Wako, Saitama, 351-0198, Japan

**Keywords:** scanning tunneling microscope, spin-polarized tunneling spectroscopy, superconducting STM tip, Yu–Shiba–Rusinov state, quantum phase transition, Zeeman effects

## Abstract

Detecting spin states of electrons at the atomic scale has been at the heart of progress in condensed matter physics. Spin-polarized scanning tunneling microscopy and spectroscopy (SP-STM/STS) has provided important insights into understanding the nature of various spin-dependent phenomena, owing to its capability to visualize energy- and spin-resolved local density-of-states with atomic resolution. This review provides an overview of recent progress in SP-STS using functionalized superconducting tips, focusing on two approaches: conventional superconducting tips and Yu–Shiba–Rusinov tips, which are formed by placing a single magnetic atom at the apex of a superconducting tip. Due to their nearly full spin polarization, both types allow for precise detection of the sample’s spin polarization. These advanced techniques will be powerful probes for pursuing emergent quantum phenomena that demand ultra-high spin sensitivity, such as the spin polarization of Majorana zero modes around vortex cores in topological superconductors.

## Teaser

We review the recent progress in spin-polarized scanning tunneling microscopy using two types of superconducting tips: (i) a conventional superconducting tip and (ii) a Yu–Shiba–Rusinov (YSR) tip. Both of them achieve unprecedentedly high spin resolution, owing to the completely spin-polarized density-of-states of these tips.

## Introduction

Spins of elections and their many-body interactions are the source of diverse emergent phenomena in solids. For complete understanding of these phenomena, it is necessary to experimentally identify the spin structure and its response to external perturbations down to atomic level. Spin-polarized scanning tunneling microscopy (SP-STM) and spectroscopy (SP-STS), which satisfies this demand, has played essential roles in uncovering various spin-dependent phenomena [1–47]. The representative examples include detection and manipulation of spins in single magnetic atoms [20–23] and molecules [24–26], identification of Ruderman–Kittel–Kasuya–Yosida interactions in magnetic atom chains [27, 28], and the observation and control of chiral and spiral spin textures [29–35] and skyrmions [36–42]. Besides the phenomena in magnetic materials, the capability of SP-STM has also been applied to investigate nature of spin density waves coexisting with unconventional superconductivity [43–45] and to examine the putative spin-polarization of Majorana zero mode at edges and vortex cores of topological superconductors [46, 47]. The recently expanding applicability of SP-STM for a wide range of phenomena demands further improvement of spin resolution in SP-STM/STS experiments.

Here, we review recent improvements in spin resolution using functionalized superconducting tips [48–59]. After a brief introduction of basic principles of SP-STM and SP-STS, we will introduce high-resolution SP-STS experiments using conventional superconducting tips and Yu–Shiba–Rusinov (YSR) tips made by attaching a single magnetic atom to a superconducting tip apex. Finally, we will discuss the advantages and future technical prospects, and potential applications.

## Principle of SP-STM and SP-STS

A fundamental quantity that describes a spin state in the aforementioned spin-dependent phenomena is a spin polarization vector composed of the spin polarization of the electronic density-of-state (DOS) [$\rho^{↑,↓}(E)$] and the direction of the spin (u):


(1) $$P(E)\equiv \frac{\rho^{↑}(E)-\rho^{↓}(E)}{\rho^{↑}(E)+\rho^{↓}(E)}u.$$

**Fig. 1.**
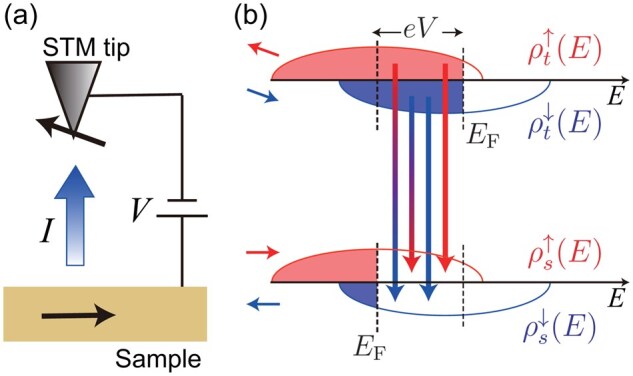
(a) Schematic illustrations of SP-STM tunneling junction. Black arrows on the tip and sample are the directions of their spins. (b) Schematic figure of possible tunneling channels. Red and blue boxes represent DOS with majority- and minority-spins of the tip (top) and sample (bottom), respectively. The shaded area in these boxes denotes the occupied states. Red and blue arrows placed at the left side of these boxes indicate directions of majority- and minority-spins, respectively. Four vertical arrows show the four possible tunneling channels of electrons.

Spin-polarized STM is able to address this quantity on a sample surface through measurements of the tunnel current flowing between an SP STM tip and the sample, separated by a vacuum tunnel barrier (Fig. 1a). When a bias voltage *V* is applied to the sample that shifts its Fermi energy ($E_{F}$) by *eV* (*e*: elemental charge), four tunneling processes occur. These are the transitions from tip’s occupied states with majority- $\rho_{t}^{↑}(E)$ or minority-spin $\rho_{t}^{↓}(E)$ to sample’s unoccupied states with majority- $\rho_{s}^{↑}(E)$ or minority-spin $\rho_{s}^{↓}(E)$, as shown by the arrows in Fig. 1b. Since the probabilities of these processes depend on the relative angle of the spins of tip and sample (θ), the total tunneling current is eventually described by


(2) $$I(V)\propto \int_{E_{F}}^{eV}dE\sum_{\sigma_{t},\sigma_{s}}\rho_{t}^{\sigma_{t}}(E)\rho_{s}^{\sigma_{s}}(E)(1\pm \text{cos}\;\theta ),$$

where, we assume the limit of small *V* and low temperature for simplicity. The majority- and minority-spin of the tip (sample) is denoted by $\sigma_{t(s)}=↑,↓$. The sign in front of the cosine is + for $\sigma_{t}=\sigma_{s}$, and is − otherwise. If we ignore the spin degree of freedom ($\sigma_{t},\sigma_{s}$), Eq. (2) is reduced to be the tunnel current for a conventional (non-spin-polarized) STM, meaning that SP-STM inherits all the functionalities of a conventional STM. These include capabilities to visualize surface structure and the local DOS (LDOS) of a sample with atomic resolution. Similar to a conventional STM, the differential conductance dI/dV(V) is related to the spin-polarized DOS of the sample as follows,


(3) $$\frac{dI}{dV}(V,r)\propto \rho_{s}^{0}(eV,r)[1+P_{t}\cdot P_{s}(eV,r)].,$$

where we also assume that tip DOS is constant against *E* for simplicity, $\rho_{s}^{0}(E,r)\equiv \rho_{t(s)}^{↑}(E,r)+\rho_{t(s)}^{↓}(E,r)$ is the total LDOS of the sample, and $P_{t(s)}$ represents spin polarization vector of the tip and sample defined by Eq. (1). Acquiring this quantity as a function of the tip location r and the voltage is known as SP-STS. If we know $\rho_{s}^{0}(E,r)$ and $P_{t}$, we can infer the spin polarization vector of the sample $P_{s}(E,r)$. An important consequence of Eq. (3) is that the larger spin polarization of the tip $P_{t}$ gives the higher spin resolution in SP-STS measurements, requiring use of a material with the larger spin polarization as a tip.

Based on this requirement, there have been various efforts to make an SP-STM tip using ferromagnetic materials [3, 6, 8]. Although the ferromagnetic tips possess relatively high spin polarization (∼40% for Fe thin layers coated on tungsten (W) tip), they create a non-negligible stray field that may locally modify the sample’s spin polarization vector around the tip. To mitigate the effect of the stray field, antiferromagnetic Cr tips have been widely used for a more reliable evaluation of the spin states in various magnetic samples [11, 53]. But, its spin polarization is as low as only ∼10%. These issues require an alternative approach for an STM tip to have a highly spin-polarized DOS as well as a small stray field.

## SP-STS using conventional superconducting tip

**Fig. 2.**
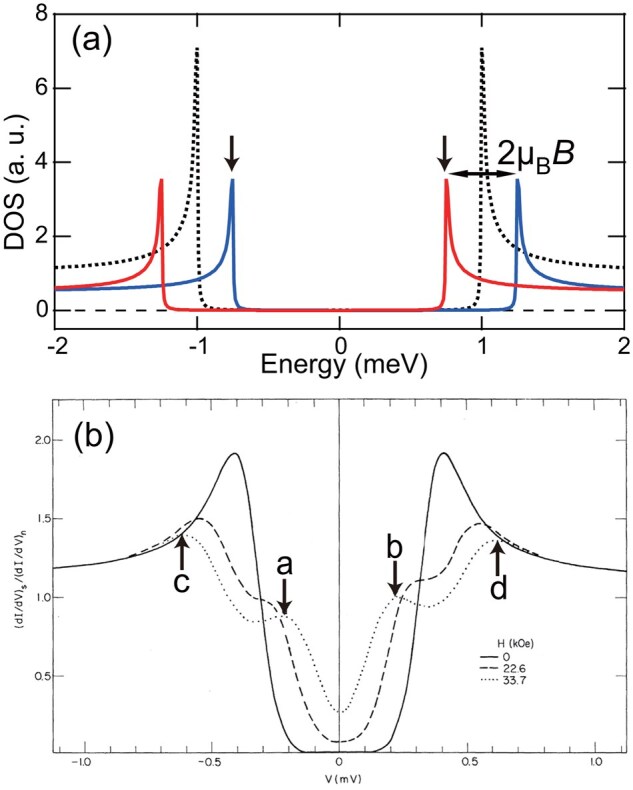
(a) DOS in a superconductor. Dashed black, solid blue, and red lines represent DOS at *B *= 0 T, spin-up and -down DOS at B≠0 T, respectively. The vertical arrows point out the edges of the inner coherence peaks where the spin polarizations are almost 100%. This figure is adapted from Ref. [50] (©2026 The Vacuum Society of Japan). (b) Tunnel conductance of an Al(superconductor)–Al_2_O_3_(insulator)–Ni(magnetic metal) planar junction where the bias voltage is applied to the Al thin film. Vertical arrows indicate the conductance at energies corresponding to Zeeman-split coherence peaks at a magnetic field of 33.7 kOe. This graph is adapted from Ref. [63] (©1971 American Physical Society).

A possible approach to realize a highly spin-polarized DOS is to use the Zeeman effect of the superconducting DOS. When a magnetic field *B* is applied to a superconductor, the coherence peaks of the superconducting gap split due to the Zeeman effect, giving rise to nearly 100% spin polarization at the gap edges as shown in Fig. 2a. By exploiting the Zeeman-split coherence peaks of the tip, we expect quite high spin resolution in spin-polarized spectroscopy. However, there are two challenges for the experimental implementation.

The first challenge is that the expected Zeeman energy is merely 58 μeV/T if the *g*-factor is 2, demanding a high energy resolution in spectroscopy. This problem can be solved by using a dilution refrigerator STM with an energy resolution as high as a few tens of μeV [60–62]. The second problem is the fragility of superconductivity against *B*, originating from the orbital pair-breaking effect. Since this effect arises from the penetration of magnetic vortices into the superconductor, it can be suppressed by choosing a proper combination of the dimensionality of the superconductor and the direction of *B*. For example, in a two-dimensional superconductor under an in-plane magnetic field, the vortices cannot penetrate into it, leading to the suppression of the orbital pair-breaking effect.

The first demonstration of this concept was done by using the planar tunnel junction composed of superconductor (aluminum thin film)–insulator (aluminum oxide thin layer)–magnetic metal (Ni) under an in-plane magnetic field [63, 64]. As shown in Fig. 2b, the tunnel conductance spectra exhibit the Zeeman splitting and asymmetric intensities at positive and negative gap edges, reflecting the spin-polarization of the DOS of Ni near $E_{F}$. Moreover, the spin-polarization could be estimated quantitatively by


(4) $$P_{\text{Ni}}=\frac{\left(dI/dV_{d}-dI/dV_{a}\right)-\left(dI/dV_{c}-dI/dV_{b}\right)}{\left(dI/dV_{d}-dI/dV_{a}\right)+\left(dI/dV_{c}-dI/dV_{b}\right)},$$

where $dI/dV_{i}$ is the conductance at point i(i=a,b,c,d) in the spectra in Fig. 2b. This method has been widely employed to estimate the spin-polarized DOS in various magnetic materials [65].

**Fig. 3.**
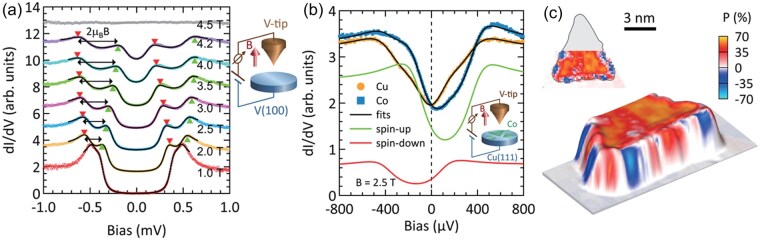
(a) The magnetic field dependence of the tunneling spectra using a superconducting V tip on the V(100) surface, which is in the normal state above 0.5 T at 15 mK. Black lines are fits based on the Maki function. Red and green triangles point out the coherence peaks of the spin-up and -down channels, respectively. (b) Tunneling spectra measured on the Cu(111) surface (orange) and the Co island (blue) at B=2.5 T. The contributions of spin-up and -down channels are shown by green and red lines extracted by fitting using the Maki function. (c) Image of the spatial variation of the absolute spin polarization around a Co island. All images are adapted from Ref. [48] (©2014 American Chemical Society).

When the apex of a superconducting tip is sharp enough to be assumed as a quasi-zero-dimensional superconductor, the orbital pair-breaking effect would be suppressed even under a magnetic field. In this case, the concept employed in the planar junction can be extended to the STM tunnel junction. Eltschka *et al.* [48, 49] experimentally implemented this idea using a sharp superconducting vanadium (V) tip, as shown in Fig. 3. The spectra exhibit clear Zeeman splitting and show that the superconductivity of the tip survives at the magnetic field as high as 4 T, which is one order of magnitude higher than the bulk critical field (Fig. 3a) [48, 49]. More importantly, Eltschka *et al.* succeeded in quantitatively evaluating the spin polarization of Co islands to be ∼65%, which is roughly twice as large as that calculated using the density functional theory calculations (Fig. 3b and c). This contradiction has been interpreted as the tip-sample distance dependence of the contributions from majority and minority states, suggesting that the STM-based spin-polarized spectroscopy could also address the envelopes of wave functions of the majority- and minority-states from the surface.

**Fig. 4.**
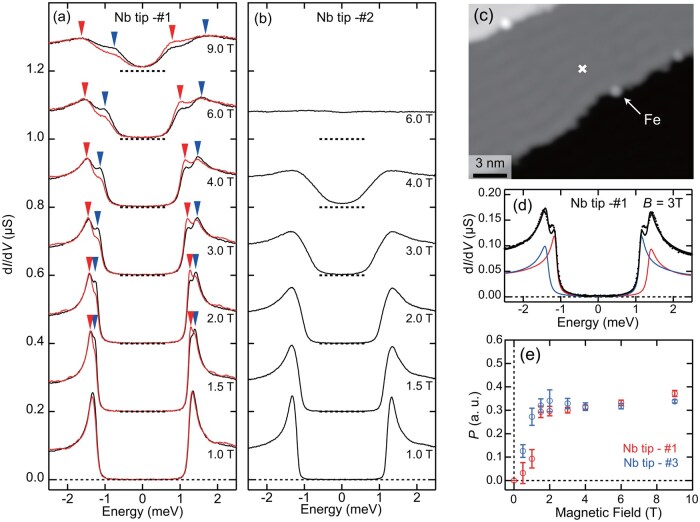
(a) The magnetic field dependence of tunneling spectra taken by using the Nb tip (#1) on Cu(111) surface (black) and on a Fe adatom (red). Red and blue triangles show the coherence peaks of the spin-up and -down channels. Locations at which the spectra are acquired are pointed out by the cross (Cu substrate) and the arrow (Fe adatom) in (c). (b) The magnetic field dependence of the tunneling spectra taken by using a Nb tip (#2) on Cu(111). (c) An STM image of the Fe adatom trapped at a step edge of the Cu(111) surface. (d) A tunneling spectrum of the Nb tip (#1) on the Cu(111) surface at B=3 T (black dots). Solid black, red, and blue lines are a fitting result using the Maki function and its spin-up and -down components, respectively. (e) Estimated spin polarization *P* of the Fe adatom trapped at a step edge for two different combinations of Nb tips and samples. The sign of the sample bias voltage *V* is flipped when converting it to energy, so that the positive energy on the spectra corresponds to the empty state of the tip. Figures (c)–(e) are adapted from Ref. [50] (©2026 The Vacuum Society of Japan).

A superconducting tip with a higher superconducting transition temperature $T_{c}$ and higher critical field $B_{c}$ expands the applicability of this technique to a wide range of phenomena. To this end, it is necessary to make a sharp enough tip made of a superconductor with a higher $T_{c}$. Recently, we focused on niobium (Nb), which has the highest $T_{c}$ in single-element superconductors [50]. To achieve the robust superconducting tip against an external magnetic field, we sharpen the Nb tip by indenting it into Nb single crystal. Figure 4a and b shows the magnetic field dependence of the tunneling spectra on a Cu(111) surface using two different tips (namely #1 and #2). Although most of the tips we examined do not show the clear Zeeman splitting as shown in Fig. 4b, some tips clearly indicate Zeeman splitting above 1.5 T, as for the tip-#1 (Fig. 4a). For all of the tips revealing the Zeeman splitting, the size of the splitting linearly increases, and the energy broadening of the coherence peaks becomes more pronounced with increasing *B*. Following the previous work by Eltschka *et al.*, we extracted the spin-up and -down components of the tip DOS by fitting the obtained spectra to the Maki function (Fig. 4d) [66–69]:


(5) $$\begin{array}{cc} \frac{dI}{dV}(V)\propto \int_{-\infty }^{\infty }dEA(E)\frac{\partial f(E-eV)}{\partial V}, & \\ A(E)=\frac{\left(1+P_{s}\right)}{2}\rho_{t}^{↑}(E)+\frac{\left(1-P_{s}\right)}{2}\rho_{t}^{↓}(E), & \\ \rho_{t}^{↑,↓}(E)=\frac{\rho }{2}\times \mathrm{sgn}(E)\times \text{Re}(\frac{u_{\pm }}{\sqrt{u_{\pm }^{2}-1}}), & \\ u_{\pm }=\frac{E\mp \mu_{B}B}{\Delta }+\zeta \frac{u_{\pm }}{\sqrt{1-u_{\pm }^{2}}}+b\frac{u_{\mp }-u_{\pm }}{\sqrt{1-u_{\pm }^{2}}}, & \end{array}$$

where Δ, Γ, and $\mu_{B}$ are superconducting gap, phenomenological energy broadening, and Bohr magneton. ζ and *b* denote the orbital pair-breaking factor and the spin–orbit scattering factor, respectively. The former gives the broadening of the coherence peaks, and the latter the mixing of the spin-up and down DOS around the gap edges. As shown in Fig. 4d, the extracted spin-up and -down DOS indicate the nearly 100% spin polarization around the inner gap edges.

To confirm the capability of spin-polarized spectroscopy, we compared the spectra taken on the Cu surface and on a Fe adatom trapped at the step edge, as shown in Fig. 4a. The spectra at the Fe adatom are signified by the asymmetric intensities at the positive and negative gap edges, reflecting the putative spin polarization of the DOS of the Fe adatom. We also estimated quantitatively the spin polarization of DOS of the Fe adatom by fitting the obtained spectra using the Maki function, and plotted them as a function of *B* for two different combinations of tips and samples. The spin polarizations saturate to be ∼35% above 1 T (Fig. 4e), ensuring the capability of quantitative estimation of spin polarization under a high magnetic field up to 9 T and its reproducibility.

A necessary condition to perform the spin-polarized spectroscopy is that the Zeeman splitting exceeds the energy broadening around the gap edges induced by the orbital pair-breaking factor ζ and the spin–orbit scattering factor *b*. The magnitude of the Zeeman splitting is determined by the *g*-factor. Since the *g*-factor is 2 in most superconductors, there is little room to enhance the Zeeman effect. On the other hand, the ζ and *b* are proportional to the square of the effective diameter at the tip apex and $Z^{4}$, respectively, where *Z* is the atomic number of the element of the superconductor. Hence, it is necessary to make the tip apex as sharp as possible and better to choose a lighter element superconductor, otherwise the reliability of the spin polarization estimated by the deconvolution using the Maki function are significantly compromised.

## SP-STS using YSR tip

**Fig. 5.**
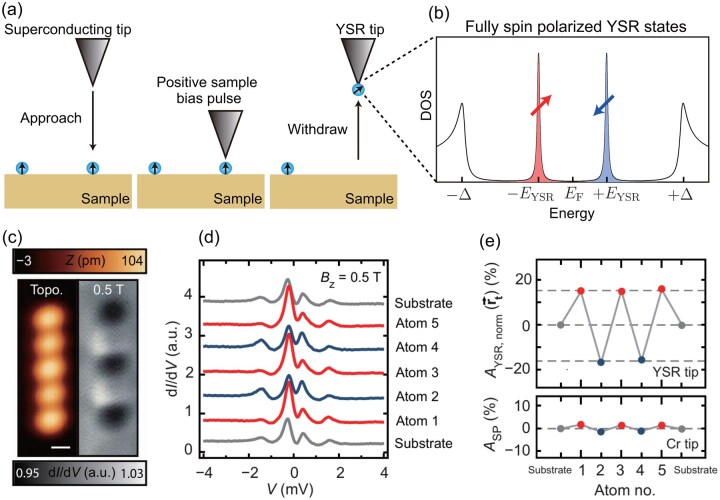
(a) Schematic illustrations of the procedure to make the YSR states at a superconducting tip apex. After approaching the tip to a magnetic adatom, a voltage pulse (usually a few V) is applied to the sample, followed by immediate retraction of the tip to the original position. (b) Schematic image of fully spin-polarized YSR states formed within a superconducting gap. (c) An STM topographic image (left) and the dI/dV maps (right) of an antiferromagnetic Mn chain on Nb(110) surface under B=0.5 T. (d) Tunneling spectra taken along the Mn chain. (e) Spatial variations of the spin polarization signals along the Mn chain using the YSR tip (top) and the conventional Cr tip (bottom). Figures (c)–(e) are adapted from Ref. [53] (©2021, The American Association for the Advancement of Science).

An alternative approach to overcome the energy broadening problem in the above-mentioned method is to exploit impurity bound states localized around a magnetic impurity in a superconductor, known as YSR states. When a magnetic impurity exists in a superconductor, the spin of the magnetic impurity induces a local exchange interaction *J* with the spins of underlying Cooper pair electrons. This interaction locally breaks Cooper pairs, thereby forming a pair of YSR bound states at symmetric energies $\pm E_{\text{YSR}}$ with in a superconducting gap [70–73] as shown in Fig. 5b. The most important feature of these states is the complete spin polarization with opposite spin species at positive and negative energies, leading to the concept that an STM tip with YSR bound states brings about the ultimate spin resolution in SP-STS.

Motivated by this concept, various experiments have been conducted to make the YSR tip and to examine its capability for the SP-STS. Huang *et al.* [51] first succeeded in creating YSR states on the apex of a superconducting V tip by picking up a magnetic impurity on a V(100) substrate, using so-called vertical atom manipulation (Fig. 5a). This technique has been widely utilized for all of the YSR tips examined so far. Intriguingly, Huang *et al.* [51] also performed tunneling spectroscopy between the YSR states at the tip end and at a magnetic impurity on the V surface (namely *Shiba–Shiba tunneling*) and found that the energy broadening of YSR states originating from their intrinsic lifetime is as small as a few hundreds of neV. This result evidences that each spin-polarized YSR state is well-isolated from the other states and suggests the potential of the YSR tip for unprecedentedly high energy resolution of sub-μeV. Immediately after these experiments, Schneider *et al.* [53] first demonstrated SP-STS on the antiferromagnetic Mn chain on Nb(110) surface using the YSR tip made by attaching Fe atoms on the Nb tip apex (Fig. 5c–e). As shown in Fig. 5c, the spatial variation of the tunneling spectra along the Mn chain reveals that the asymmetry of the YSR peak intensities between positive and negative energies alternatively changes atom by atom, demonstrating the successful detection of the antiferromagnetic spin structure of the Mn chain (Fig. 5d). More importantly, the spin polarized signals using the YSR tip are one order of magnitude greater than those for the Cr tip ($P_{t}∼$9%). This attests to the extremely high spin sensitivity of the YSR tip.

**Fig. 6.**
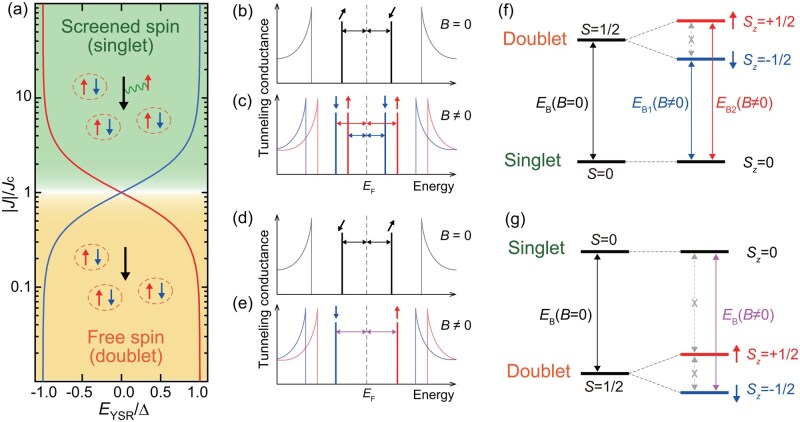
(a) Phase diagram of YSR states as a function of the exchange coupling *J*. Red and blue lines represent $E_{\text{YSR}}$. (b) and (c) Schematics of tunneling spectra YSR states for screened-spin ground state at B=0 (b) and B≠0 (c). (d) and (e) Same as (b) and (c) but for the free-spin ground state. (f) and (g) Energy diagrams of Zeeman effects on YSR states for screened-spin and free-spin ground states, respectively. Figures (b)–(g) are adapted from Ref. [54] (©2022 American Physical Society).

Even though an YSR tip achieves high spin resolution, it is also noteworthy that the YSR state generally undergoes a quantum phase transition (QPT) at a critical value of the exchange coupling $J_{c}$, which affects the spin state of the YSR tip [74–78] (Fig. 6a). Here, we briefly review the nature of the QPT of YSR states. When the *J* is large enough ($J>J_{c}$), the impurity spin is screened by itinerant electrons (Kondo screening), being known as the screened-spin ground state [79–83]. Assuming $S_{\text{imp}}=1/2$ for simplicity (the same argument also applies for higher impurity spin) [54], the screened-spin ground state is a spin-singlet (S=0). The YSR states are the lowest excited states of a single spin-1/2 electron from the ground state. Therefore, for the spin-singlet ground state, its excited state should be a spin-doublet (S=1/2), yielded when the ground state loses (acquires) an electron whose spin is parallel (antiparallel) to the impurity spin. These spin-dependent excitation processes are a source of perfect spin polarization of the YSR state. In the opposite case for $J<J_{c}$, the impurity spin is approximately isolated (namely the free-spin ground state), giving rise to the spin doublet ground state (S=1/2). In this case, the excited electrons have opposite spin species compared to those for the screened-spin regime. Consequently, through the QPT, the spin of the YSR states is inverted at $J=J_{c}$ (Fig. 6a, b, and d). The reversal of the YSR states demands unambiguous identification of the ground state for a given YSR tip, before using it for the SP-STS experiments, as discussed in Ref. [53].

The differences between these ground states become pronounced in Zeeman effects on the YSR states [84, 85]. For the screened-spin ground state, the applied magnetic field *B* splits the doublet excited state by the Zeeman effect, whereas the singlet ground state remains intact (Fig. 6f). Therefore, the YSR peaks in the tunneling spectrum show Zeeman splitting (Fig. 6c). On the other hand, for the free-spin ground state, Zeeman splitting occurs at the spin doublet ground state (Fig. 6g), causing the new ground state under the magnetic field to be the Zeeman split lower energy state. If the electron temperature is well below the Zeeman-splitting energy, no thermally excited quasiparticles in the Zeeman-split higher-energy state. Hence, only the excitation from the Zeeman-split lower energy state contributes to the YSR peak, resulting in a Zeeman shift rather than the Zeeman splitting in the spectrum. Eventually, this qualitative difference of the Zeeman effects would be a crucial tool to discriminate between the ground states.

**Fig. 7.**
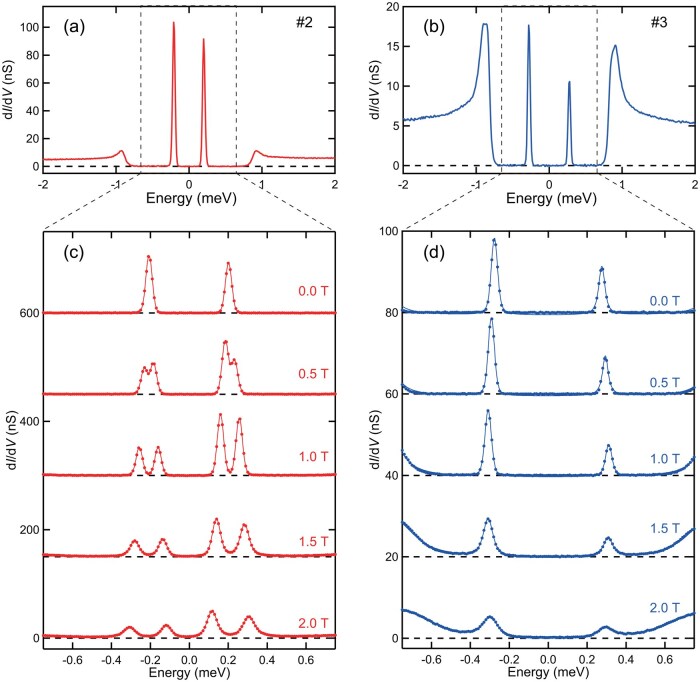
(a) A tunneling conductance dI/dV spectrum of the tip #2 showing the YSR peaks at $E_{\text{YSR}}=\pm$207 μeV within the superconducting gap Δ∼900  μeV. (b) A dI/dV spectrum of the tip #3 where the YSR peaks appear at $E_{\text{YSR}}=\pm$278 μeV. (c) Magnetic-field dependence of the YSR peaks in #2. Each YSR peak shows Zeeman splitting, signifying the screened-spin ground state. (d) Magnetic-field dependence of the YSR peaks in #3. Each YSR peak shifts to higher |E| but does not split, indicating the free-spin ground state. The sign of sample bias voltage V is flipped when converting it to the energy, so that the positive energy on the spectra corresponds to the empty state of the tip. All figures are adapted from Ref. [54] (©2022 American Physical Society).

To demonstrate the discrimination of these ground states by detecting Zeeman effects, we examine the magnetic field dependence of the tunneling spectra of two YSR tips (Fe atom on Nb tip apex) in different ground states on a non-magnetic Cu(111) surface (Fig. 7a and b) [54]. At B=0 T, both of them represent one pair of YSR states near ±250 μeV, and their ground states are indistinguishable. Under a magnetic field, each YSR peak in #2 splits into two peaks (Fig. 7c), signifying the screened-spin state. By contrast, the YSR peaks in #3 do not split but merely shift (Fig. 7d), being consistent with the free-spin ground state. These results imply that the Zeeman effects of the YSR states provide an unambiguous determination of the ground states.

**Fig. 8.**
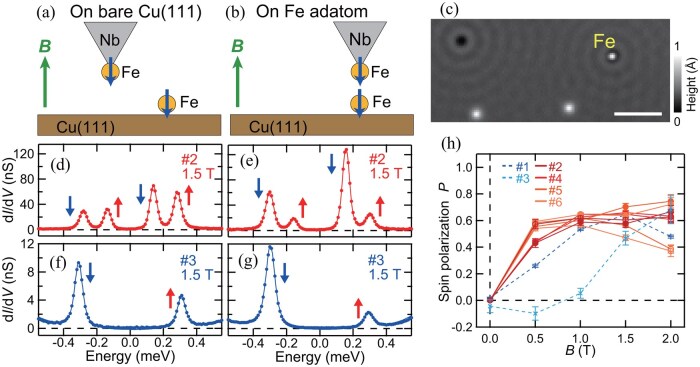
(a) and (b) Schematic illustrations of the experimental configurations used for the YSR-state-based spin-polarized STM/STS measurements. (c) An STM topographic image around the Fe adatom on which the spectroscopic measurements have been carried out. Reference spectra on the bare Cu(111) surface were taken at clean locations at least 5 nm away from the Fe adatom. The scale bar denotes 5 nm. (d) and (e) Tunneling spectra of the tip #2 (screened-spin ground state) on the bare Cu(111) surface and on the Fe adatom, respectively. (f) and (g) Tunneling spectra of the tip #3 (free-spin ground state) on the bare Cu(111) surface and on the Fe adatom, respectively. A magnetic field of 1.5 T was applied perpendicular to the Cu(111) surface. (h) Magnetic-field dependence of the spin polarizations estimated using various tips and different pairs of the YSR states. The solid and dashed lines denote the results of the tips with the screened-spin and free-spin ground states, respectively. The filled and open circles represent the data obtained for the tips with the screened-spin ground state using the Zeeman-split YSR states in the filled and empty states, respectively. The sign of the sample bias voltage V is flipped when converting it to the energy, so that the positive energy on the spectra corresponds to the empty state of the tip. All figures are adapted from Ref. [54] (©2022 American Physical Society).

We also examined the capability of spin-polarized spectroscopy for both ground states on a magnetic Fe adatom on Cu(111). Figure 8d–g compares the tunneling spectra taken on the Cu(111) surface and the Fe adatom using the tips #2 and #3, which have the screened- and free-spin ground states, respectively. In both cases, the intensities of the field-lowered peaks are enhanced on the Fe adatom, whereas those of the field-lifted peaks are suppressed compared to their counterparts on the Cu(111) surface, evidencing the spin polarization of the YSR states. We also evaluated the spin polarization of the tunneling signals *P* for the seven different YSR tips, where *P* is defined as


(6) $$P\equiv \frac{\left(W_{-}^{\text{Fe}}/W_{-}^{\text{Cu}})-(W_{+}^{\text{Fe}}/W_{+}^{\text{Cu}}\right)}{\left(W_{-}^{\text{Fe}}/W_{-}^{\text{Cu}})+(W_{+}^{\text{Fe}}/W_{+}^{\text{Cu}}\right)}.$$

Here, the *W* is the weight of the YSR peak, the superscripts denote the tunneling locations, and the subscripts − and + indicate the field-lowered and field-lifted YSR peaks, respectively. Even though the seven tips have different YSR states, the obtained *P* takes a similar value of ∼0.6 at high fields, suggesting the complete spin polarization of the YSR states and the capability of quantitative measurements of the sample’s spin polarization. It is also important to note that the necessary energy resolution of STM for YSR-based SP-STS varies depending on the ground states of the YSR states. For the free-spin ground state, the required energy resolution is better than 2$E_{\text{YSR}}$, because spin-up and -down YSR states are separated by 2$E_{\text{YSR}}$. On the other hand, for the screened-spin ground state, the energy resolution should be better than Zeeman splitting; otherwise, spin-up and -down YSR states caused by the Zeeman splitting mutually overlap, making the spin-polarized measurements ambiguous.

**Fig. 9.**
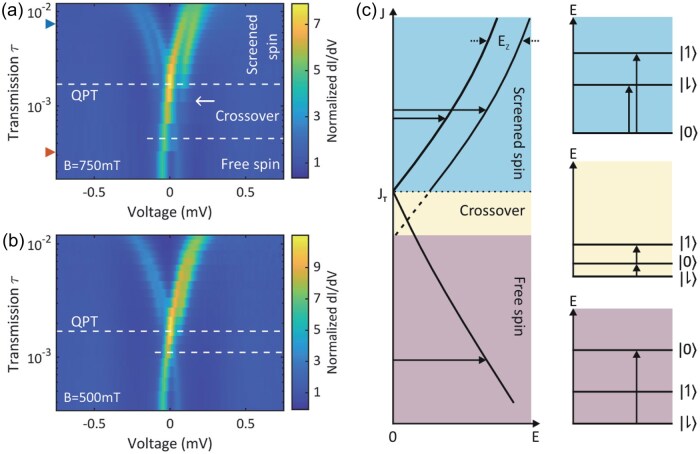
(a) A color map of tunneling conductance spectra at B=750 mT as a function of transmission of the tunneling junction τ, which is controlled by changing the tip-sample distance. (b) Same as (a) for 500 mT. The tunnel junction is composed of a field-induced normal metal V and an YSR tip (a magnetic adatom on the V tip). (c) Schematics of the energy level structures of YSR states under a magnetic field in different ground states across the QPT. All figures are adapted from Ref. [56] (©2024 Springer Nature).

A full understanding of the nature of the QPT requires systematic surveys of YSR states as a function of the exchange coupling *J*. For the YSR states of a single magnetic atom and molecule on a superconducting substrate, it has been established that the QPT can be controlled by varying the tip-sample distance through the tip-induced change in *J* [86–88]. Karan *et al.* [56] extended this technique to the YSR tip and investigated how the tunnel spectra change from Zeeman shifting in the free-spin regime to Zeeman splitting in the screened-spin regime near the critical point. Intriguingly, there is a crossover regime where the Zeeman splitting starts evolving even in the free-spin regime (Fig. 9a and b). This Zeeman splitting in the crossover regime has been interpreted as the two possible excitations emerging when the spin-singlet excited state (S=0) places between the Zeeman split spin-doublet ground states (middle region in Fig. 9c). These findings will be essential to not only control the YSR ground states but also accurately determine the ground state when the YSR states lie very close to zero energy.

Controlling the spin direction of the YSR tip is also important to obtain the complete three-dimensional information of the spin states. Recently, it was demonstrated that the spin direction at a single Cr atom on a Nb tip can be controlled three-dimensionally by reasonably small vector magnetic fields (∼1 T), indicating the approximately paramagnetic nature (with small magnetic anisotropy) of the Cr spin on the Nb tip apex [57]. In general, such tunability of the spin direction is mainly governed by the magnetic anisotropy that depends on various factors, including the element and absorption site of the magnetic atom, and spin–orbit coupling of the host superconductor. Therefore, it is required to establish a way to identify the magnitude and the direction of the magnetic anisotropy of a given YSR tip.

## Summary and future prospects

In this review, we introduced the recent development of high spin resolution spin-polarized spectroscopy using a superconducting tip with Zeeman split coherence peaks and an YSR tip. Owing to their complete spin polarization, both types of tips bring about quantitative detection of the spin polarization of the sample with exceptionally high spin resolution. Nevertheless, there remain several challenges. For superconducting tips, it is essential to sharpen the tip apex as much as possible. However, the yield of sufficiently sharp tips using the preparation method introduced here remains very low. To address this issue, it might be helpful to combine with micro-fabrication techniques such as focused ion beam processing. Another drawback of the superconducting tip is that spin-polarized spectroscopy is impossible at zero magnetic field, in principle. In contrast, the YSR tips would enable the high spin resolution spectroscopy even at zero magnetic field, because the YSR state remains fully spin-polarized at zero field. However, the strength and orientation of the magnetic anisotropy of the magnetic adatom, which govern the spin direction of the YSR state at zero magnetic field, have to be determined. If these technical challenges can be overcome, the spin-polarized STM methodology introduced here holds great promise for a wide range of applications. These include detecting the spin polarization of Majorana quasiparticles expected to emerge at the vortex cores of topological superconductors, and probing the spin polarization of Landau levels in topological insulators.
